# Editorial: Poultry Coccidiosis: Strategies to Understand and Control

**DOI:** 10.3389/fvets.2020.599322

**Published:** 2020-10-23

**Authors:** Anderson Ferreira da Cunha, Elizabeth Santin, Michael Kogut

**Affiliations:** ^1^Laboratory of Biochemistry and Applied Genetics, Department of Genetics and Evolution, Center for Biological and Health Sciences, Federal University of São Carlos, São Paulo, Brazil; ^2^Microbiology and Avian Pathology Laboratory, Department of Veterinary Medicine, Federal University of Parana, Parana, Brazil; ^3^Southern Plains Agricultural Research Center, U.S. Department of Agriculture-USDA, Agricultural Research Service, College Station, TX, United States

**Keywords:** molecular diagnosis, poultry health, vaccine, *Eimeria*, coccidiosis

Poultry coccidiosis is a parasitic intestinal infection, caused by several species of *Eimeria*. Coccidiosis is the most prevalent disease in broilers with worldwide economic losses estimated to be up to US$ 3 billion per year. Understanding the relationship between nutrition and *Eimeria* infection is an important component of the growth and sustainability of poultry worldwide. Recognizing the role of nutrition in coccidial infections as part of new technologies to control disease pathogenesis should be a component of future control strategies. The papers presented in this collection address these findings and other new research in the control of coccidiosis.

The first step in discovering new control measures against coccidiosis is understanding the host:*Eimeria* relationships during the initial steps of infection. López-Osorio et al. presented new data describing the development of pathogenicity and immune response triggered by the early stages of the *Eimeria* life cycle (*E. tenella, E. necatrix, E. acervulina, E. maxima, E. brunetti, E. mitis*, and *E. praecox*).

More accurate methods to measure the different parameters involved in the performance of the birds under *Eimeria* challenge are of primary importance. The I See Inside (ISI) methodology was recently developed and described by Sanches et al. to evaluate basal and infectious enteritis in chickens. The results showed the feasibility of ISI as an *in-situ* method that describes the pathogenic mechanisms of inflammation associated with losses in performance during subclinical *Eimeria* and necrotic enteritidis infections.

Administration of ionophores has long been employed as the best effective method to control *Eimeria* infections. However, ionophores are classified as antibiotics and current political and societal pressure has resulted in the reduction and/or the banning of the use of these molecules in livestock production. Therefore, the use of dietary nutrients and feed additives as a means of controlling *Eimeria* infection and improving the broiler gut health were discussed and focused on answering some essential questions in the field. Exploring this area, Bortoluzzi et al. focused on how micro-minerals and their absorption by the gastrointestinal tract (GIT) could have an important role in the modulation of the intestinal physiology, immunity, and microbiology of broiler chickens during *Eimeria* infection. They showed the beneficial effects, increased performance and attenuated the inflammatory response when feeding Zn to broilers infected with coccidia and *Clostridium perfringens*. Additionally, Zn modulates the ileal microbiota thus improving the gut health of broilers. Further, Cu and Mn provided as feed additives during *Eimeria* infection were also shown to improve feed conversion and modulate the immune response during the infection.

Similarly, Kiarie et al. focused on reviewing the role of feed enzymes and yeast derivatives in modulating coccidial infection. The authors discussed the use of these additives as an alternative and/or complementary strategy for the control of the infection. They cited several papers in which the use of whole yeast or its derivatives as nucleotides or cell wall-associated with feed enzymes enhanced cellular and humoral immunity which would be of utmost importance for increasing the effectiveness of coccidial vaccines.

Two original research papers about feed additives were also published here. Stefanello et al. discussed the use of a blend of protected organic acids and essential oils to improve the health of broilers challenged with *Eimeria* spp. and with *Clostridium perfringens*. The blend of protected organic acids and essential oils improved growth performance, nutrient digestibility, and intestinal health in the treated animals better than growth promoter (AGP) and could be an excellent alternative in AGP-free programs. Similarly, Calik et al. showed the improvement in the immune response followed by a modification in the intestinal morphology and increase in the performance of broiler chickens challenged with coccidiosis when fed with direct feed microbial (DFM) dietary additive. Chickens treated with the additive showed an increase in body weight gain and a reduction in the lesions at the duodenum and jejunum and an increment of the ileal villus area. An increase in the expression of IFN-γ and IL-1β were also observed in the ileum, showing that the feed with microorganisms could be a promisor technique to improve the health of broilers.

Vaccination as the preferred method to prevent the infection was also discussed in a review of Soutter et al.. Further, the development of standard protocols to evaluate vaccine efficacy and the improvement of performance and nutrient utilization by birds is required. Gautier et al., describing the timing and magnitude of coccidiosis vaccination and its influence of growth performance and nutrient utilization during the various stages of *Eimeria* cycling. Coccidial vaccination impaired the feed conversion ratio (FCR) but did not change the body weight gain (BWG) and the feed intake (FI).

Taken together the papers published in this Research Topic showed several important results that could be used for the avian industry and highlight the importance of new studies in the exciting area of poultry coccidiosis.

## Author Contributions

All authors listed have made a substantial, direct and intellectual contribution to the work, and approved it for publication.

## Conflict of Interest

The authors declare that the research was conducted in the absence of any commercial or financial relationships that could be construed as a potential conflict of interest.

